# Polymer Hybrid Nanocomposites Based on Homo and Copolymer Xlpe Containing Mineral Nanofillers with Improved Functional Properties Intended for Insulation of Submarine Cables

**DOI:** 10.3390/polym14173444

**Published:** 2022-08-23

**Authors:** Leszek Resner, Pawel Lesiak, Iman Taraghi, Agnieszka Kochmanska, Pawel Figiel, Elzbieta Piesowicz, Marek Zenker, Sandra Paszkiewicz

**Affiliations:** 1 Department of Materials Technologies, West Pomeranian University of Technology, Al. Piastów 19, 70-310 Szczecin, Poland; 2 Tele-Fonika Kable S.A., Factory in Bydgoszcz, Bydgoszcz ul. Fordońska 152, 85-957 Bydgoszcz, Poland; 3 Department of High Voltage and Power Engineering, West Pomeranian University of Technology, ul. Sikorskiego 37, 70-313 Szczecin, Poland

**Keywords:** polymer hybrid nanocomposites, XLPE, HNT, cloisite, submarine power cables, cable insulations, water treeing effect

## Abstract

Cross-linked polyethylene (XLPE) is one of the most popular insulation materials used in the production of medium and high voltage cables (MV, HV). This article presents the results of research carried out on two types of commercially used insulation materials, modified with the addition of organophilic phyllosilicate (CLOISITE C20A)and halloysite nanotubes (HNTs). The influence of fillers on the mechanical properties of insulating materials is discussed as a potential mechanism for increasing their resistance to the phenomenon of water-tree. SEM and XRD analyses were performed to investigate the morphology and DSC for comparing phase transitions. Mechanical and functional properties for different concentrations of nanofillers, such as their hybrids, were also investigated.

## 1. Introduction

The most crucial element in cables designed for medium and high voltage transmission networks is the insulation. If the cable has no manufacturing defects, insulation material properties determine the service life and operational safety of the electricity transmission line [1]. Since the 1960s, crosslinked polyethylene (XLPE) has been used as an insulating material because it has very good electrical properties, and mechanical and thermal strength, which makes it resistant to aging [2,3]. The most dangerous phenomenon which leads to reduce the lifespan of the XLPE and, as a result, loss of dielectric parameters by cable insulation, is water tree growth. This process is created under the influence of an electric field and moisture, and it is initiated in the areas where defects occur, such as e.g., micro-cracks [4,5]. It has been proven that this phenomenon occurs only when the relative humidity in cable insulation exceeds 70%, and despite the use of the different types of water barriers in cable construction, it is not always possible to prevent water migration to the cable insulation [6]. Especially when they are submarine cables and in addition designed for special applications, such as floating wind farms, so-called dynamic cables [7]. The insulation of such submarine cables is particularly vulnerable to mechanical damage that may occur in the production process, and at a later stage during installation and even operation [8,9]. Dynamic submarine cables are made of twisted three power cores, armored with one or, in the case of dynamic cables, with several layers of steel wires [10]. Stress during installation at the seabed and then continuous bending of cables due to the floating of wind turbines on waves may cause micromechanical damage to the insulation, initiating the water tree effect [11,12]. Therefore, a lot of research has been done to modify XLPE materials in order to improve their mechanical properties [13].

Modification of polymer materials by mineral nanofillers, such as clays and other fillers, makes the polymer nanocomposites suitable for diverse applications: in the packing industry, in the car industry because of the heat resistance, as a lubricant in petroleum extraction, and as barrier materials for instance in the cable industry. To achieve better mechanical properties, adequate interfacial clay–polymer bonding is needed. According to the literature, different kinds of layered silicates have been used in the synthesis of PCNs; the most common one is montmorillonite, which is a 2:1 aluminosilicate composed of an octahedral aluminum oxide layer sandwiched between two tetrahedral silicon oxide layers. Substitution occurs in the tetrahedral and octahedral layers. Regarding the octahedral layer, aluminum ions (Al^3+^) could be substituted by magnesium (Mg^2+^) and iron ions (Fe^2+^). In the tetrahedral layers, Al^3+^ could replace Si^4+^. In both cases, the substitution will lead to an overall negative charge, which will be compensated by cations such as Na^+^ and Ca^2+^ existing in the region between the clay layers known as interlayer regions. Cloisite^®^ 20A is a montmorillonite clay, wherein the tallow-triethanol-ammonium ion replaces the Na^+^ ions. When the clay plates are completely dispersed into the polymer matrix, this is referred to as full exfoliation. This kind of composite has shown improvement in the properties because of the increased reinforcement. Intercalated systems that are characterized by the insertion of polymer between the plates of clay followed by an increase of the spacing between the plates is called gallery spacing [14,15]. The changes in the gallery spacing are measured by X-ray diffraction (XRD). In the case of an exfoliated system, the reliable technique is transmission electron microscopy. Halloysite nanotubes (HNT) have a high amount of 1D nanotubular structures with a high length-to-diameter ratio and low hydroxyl group density on the surface. The HNT contains nanotubes and nanoplatelets of halloysite and similarly to montmorillonite, it has two layers of aluminosilicate [14]. The HNTs contained in this nanoclay offer numerous benefits because of their high mechanical strength, thermal stability, and biocompatibility [16].

There have been several publications that show the properties of LDPE or HDPE/clay nanocomposites which were affected by the incorporation of mineral nanofillers. Among them, are Mallayan et al. [17] who analyzed the performance of LDPE/clay nanocomposites. The inclusion of nano montmorillonite (MMT) clay in LDPE material has significantly increased the contact angle, corona aging resistance, water droplet initiated corona inception voltage, and surface discharge inception voltage of the composites [17]. Said et al. [18] studied linear low-density polyethylene (LLDPE)/clay nanocomposites with different clay contents prepared by melt intercalation using two different compatibilizers: maleic anhydride grafted styrene–ethylene–butylene–styrene and maleic anhydride grafted polyethylene (PE-g-MA). In this paper, the effects of clay and compatibilizer fractions and the type of compatibilizer on the structure, permeability, and barrier properties of the nanocomposite films were studied. The results revealed that not also clay, but the compatibilizer participated in decreasing the permeability of the film [18]. Beesetty et al. [19] investigated the influence of clay on the mechanical properties of HDPE. The results show that the solid particle reinforced composites have a higher density and modulus as expected. Moreover, there are some studies on the effects of clay and HNT nanofillers on the physical properties of the XLPE matrix [19]. Li et al. [20] have blended the organic montmorillonite (OMMT) particles treated by two organic intercalants (octadecyl quaternary ammonium salt and double octadecyl benzyl quaternary ammonium salt) with crosslinked polyethylene to prepare the nanocomposites. Jose and Thomas [21] have investigated the mechanical and thermal properties of Alumina-clay nanoscale hybrid filler assembling in cross-linked polyethylene-based nanocomposites. All the nanocomposites exhibited improved mechanics, and the ternary hybrid composite of Al_2_O_3_ and clay in a 1:1 ratio showed the highest tensile strength (100% increase) and Young’s modulus (208% increase), followed by Al_2_O_3_:clay = 2:1, binary systems of XLPE-Al_2_O_3_ and XLPE-clay [21]. In another study, Jose et al. [22] investigated the wetting properties and surface energy characteristics of cross-linked polyethylene (XLPE) with different nanofillers (such as Al_2_O_3_, SiO_2_, TiO_2_, and clay) using contact angle measurements with water and dimethyl sulfoxide (DMSO) as solvents. Comparing interfacial energy and interaction parameter, and all other properties XLPE/Al_2_O_3_ nanocomposite is the best candidate having higher non-wetting properties and could succeed in fabricating a non-wettable surface of nanocomposite from a wettable surface of XLPE by the addition of surface treated inorganic nanofiller [22]. Furthermore, Shared et al. [23] have studied the application of surface-modified XLPE nanocomposites for electrical insulation-partial discharge and morphological study. Additionally, Kavitha and Balachandran [24] have indicated the performance of nano-reinforced (nanoclay) XLPE for high-voltage insulation applications with a particular focus on dielectric characteristics, treeing behavior, and mechanical properties. The mechanical properties of the nanocomposite (tensile strength and modulus) were increased due to polymer and nanofiller interactions [25].

This study aims in comparing the influence of two types of mineral nanofillers, organophilic phyllosilicate (C20A) and halloysite nanotubes (HNT), and their hybrid system, on the properties of two types of commercially available insulation materials, homo and copolymer XLPE. The reference materials were the commercially available grades of XLPE (LS4201R), not resistant to the phenomena of water treeing, hereinafter referred to as PELS, and resistant to the phenomenon of water treeing LC8205R, hereinafter referred to as PELC. Both types of insulation materials were modified with both additives in concentrations of 2.5%, 5.0%, and 7.0% and a 50/50 C20A hybrid with HNT in concentrations of 5.0% and 7.0%. In the paper, the morphology, mechanical and utilitarian properties of these modified materials were examined, and the results were discussed in the context of their influence on the service life of the obtained insulating material in terms of submarine cables application.

## 2. Sample Preparation and Characterization Techniques

### 2.1. Materials

Two types of commercially available insulation materials have been used as polymer matrices: (1) LS4201R (Borlink TM, Borealis, Vienna, Austria) is a cross-linkable natural polyethylene compound, specially designed for insulation of power cables; with the density (Base Resin) of 922 kg/m^3^, and MFR (190 °C/2.16 kg) 2.0 g/10 min; (2) LC8205R (Borlink TM, Borealis, Vienna, Austria) is a ready-to-use natural co-polymer compound with special features: it provides superior electrical performance (polymer WTR XLPE) meeting the most stringent wet aging requirements, it offers excellent scorch resistance, long production runs, and high line speed potential, with density (Base Resin) of 924 kg/m^3^ and MFR (190 °C/2.16 kg) 3.0 g/10 min.

**Figure 1:**
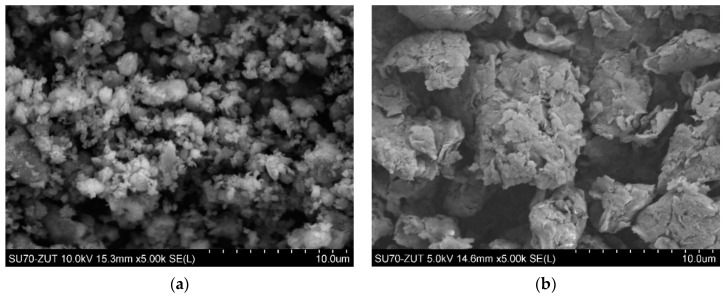
SEM micrographs of the mineral nanofillers as received: (**a**) HNT; (**b**) C20A.

**Figure 2:**
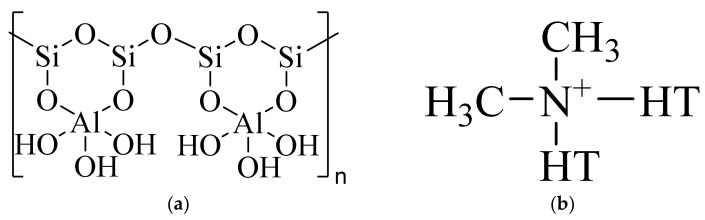
Scheme showing the structures of: (**a**) HNT; (**b**) C20A2.2.

Mineral nanofillers used in this study were as follows: halloysite nanotubes (HNT) (under the trademark “Dunino”) in form of a gray-brick color powder containing rod-shaped HNTs of diameter 100–140 nm and length 1–5 μm, bulk density 450–600 g/dm^3^ was obtained from Intermark (Gliwice, Poland) (Figure 1a); the organically modified Cloisite^®^ 20A (Southern Clay Products) was used as received, in the form of white powder and size of particles of D50: <10 μm, with a bulk density of 350 kg/m^3^, the distance between the lamellas (XRD, d001): 2.7 nm (Figure 1b). Schematic structures of both nanofillers used in tested nanocomposites are shown in Figure 2.

### 2.2. Sample Preparation

In order to obtain the samples, it was necessary to start with the preparation of granules from the base material, PELS, and PELC with the addition of nanofillers, i.e., HNT and C20A. Each of the materials was previously dried in a dryer under a dynamic vacuum. The granulate was prepared on a counter-rotating twin-screw extruder equipped with a set of gravimetric feeders, which dosed the set proportion of the blend components. To obtain a good dispersion of nanoparticles, all granulates were extruded twice, to obtain shear forces comparable to devices used in the cable industry.

The temperature of the extrusion was set based on the TDS of the material as well as the DSC measurement of the granulates of PELS and PELS to avoid cross-linking of the polymer during processing. Thus, the following parameters were determined for extruding PELS and PELC-based nanocomposites: zone 1 (feed zone): 90 °C, zone 2: 95 °C, zone 3: 95 °C, zone 4: 110 °C, zone 5: 115 °C, zone 6: 120 °C, zone 7: 120 °C, zone 8 (die) 135 °C. The rotational speed of the screws of 40 rpm. Yield: 2.5 kg/h. While for extruding PELS and PELS-based nanocomposites the following parameters were determined: zone 1 (feed zone): 95 °C, zone 2: 95 °C, zone 3: 100 °C, zone 4: 115 °C, zone 5: 120 °C, zone 6: 125 °C, zone 7: 130 °C, zone 8 (die) 135 °C. The rotational speed of the screws of 40 rpm. Yield: 2.5 kg/h.

Both series of nanocomposites based on PELS and PELC were pelletized, and injection molded using Boy 15 (Dr BOY GmbH&Co., Neustadt, Germany) to obtain dumbbell shape samples, type A3, for tensile, density, water absorption, and hardness measurements. Samples before the injection molding were dried for 24 h under a vacuum at the temperature of 60 °C. The following parameters were used: injection temperature: 135 °C, injection pressure 75 MPa, mold temperature 30 °C, holding down pressure of 20 MPa for 4 s, and cooling time of 4 s.

Samples for electrical resistivity measurements were formed at the temperature of 135 °C and under the pressure of 5 bar (for 2 min) and 10 bar (for 1 min) by compression molding (Colin P200E, Dr COLLIN GmbH, Ebersberg, Germany) to the form of polymer foils with the thickness of 1 mm. The thickness of polymer foils was measured with a Micrometer Model No. 293–521 from Mitutoyo. Five measurements were taken for each sample, with an experimental error of ±0.001 mm. The thickness is an average value.

### 2.3. Characterization Techniques

The dispersion of nanoparticles in the polymer matrix, as well as nanofillers as received were investigated by field emission scanning electron microscopy (FE-SEM Hitachi SU-70, Tokyo, Japan). The nanocomposites’ samples were cryofractured in liquid nitrogen, and then vacuum coated with a thin gold-palladium film (using JEOL JEE-4X, Japan) before the test. In SEM studies, secondary electrons (notation SE in photographs) images using acceleration voltage 5 kV were acquired.

The samples’ structure was analyzed by a differential scanning calorimeter (DSC). Measurements were carried out with a DSC 204 F1 Phoenix (Netzsch) at a heating-cooling-heating cycle at the rate of 10 °C/min, in the temperature range of −70–250 °C. The first cooling and second heating scans were used to determine the melting and crystallization peaks, respectively. The first heating was done to check if there were any cross-linking peaks.

The X-ray diffraction (XRD) analysis of the samples was conducted using a Panalytical X’Pert diffractometer (Malvern Panalytical, Malvern, UK) operating at 40 Kv and 40 mA with CuKα radiation (λ = 0.154 nm). The samples were scanned from 2θ = 4° to 70° with a step of 0.02°.

Density was studied by hydrostatic weighing method with the use of AGN200C., at 20 °C, according to ISO 1183 standard. Before measurements, the hydrostatic balance was calibrated using standards of known density. Measurements were repeated six times for each sample.

The tensile properties of the blends compositions were measured according to ISO 527 using Autograph AG-X plus (Shimadzu, Kyoto, Japan) tensile testing machine (class 1.0 according to EN 10002-2, ISO 7500-1, BS 1610, ASTM E4, JIS B7721), equipped with a 1 kN Shimadzu load cell, an optical extensometer (class 0.5 according to ISO 9513), and the TRAPEZIUM X computer software (version 1.4.5, Shimadzu, Kyoto, Japan), operated at a constant crosshead speed of 1 mm/min. Measurements were performed at room temperature on the dumbbell samples with a grip distance of 30 mm. Seven measurements were conducted for each dumbbell-shaped sample (type A3), and the results were averaged to obtain a mean value.

Hardness was measured using a Zwick 3100 Shore D tester (Zwick GmbH, Ulm, Germany). Each reported value was the mean of ten independent measurements.

Additionally, water absorption tests were performed in cold and boiling water. The measurement was carried out according to the procedures recommended in ASTM D570. First, the dumbbell shape samples were dried at 55 °C for 24 h. Subsequently, samples were cooled to room temperature and weighed. The measurement of water absorption in boiling water was carried out for 30 min, after that the samples were cooled in distilled water for 15 min. The water absorption in cold water was investigated by immersing samples in distilled water at 23 °C for 24 h. The water on the surface of the samples was removed with filled paper. After that, samples were weighted. Each reported value is an average of three test specimens.

The thermo-oxidative stability of the blend compositions used in this paper was evaluated by thermogravimetry (TGA 92-16.18 Setaram, Caluire, France) using the system measuring simultaneously TG-DSC. Measurements were carried out in an oxidizing atmosphere (i.e., dry, synthetic air (N_2_:O_2_ = 80:20 vol.%)). The study was conducted at a heating rate of 10 °C/min in the temperature range of 20–700 °C.

The electrical resistivity of the prepared nanocomposites was estimated based on the volume resistivity measurements which were performed according to the PN-EN ISO 3915 and PN-88/E-04405 standards. Keithley Electrometer 6517A (Keithley Instruments, Inc., Cleveland, OH, USA) together with a set of Keithley 8009 was used for conducting the measurements of the volume resistivity of the 1 mm thick nanocomposite films.

## 3. Results and Discussion

### 3.1. Morphology

**Figure 3:**
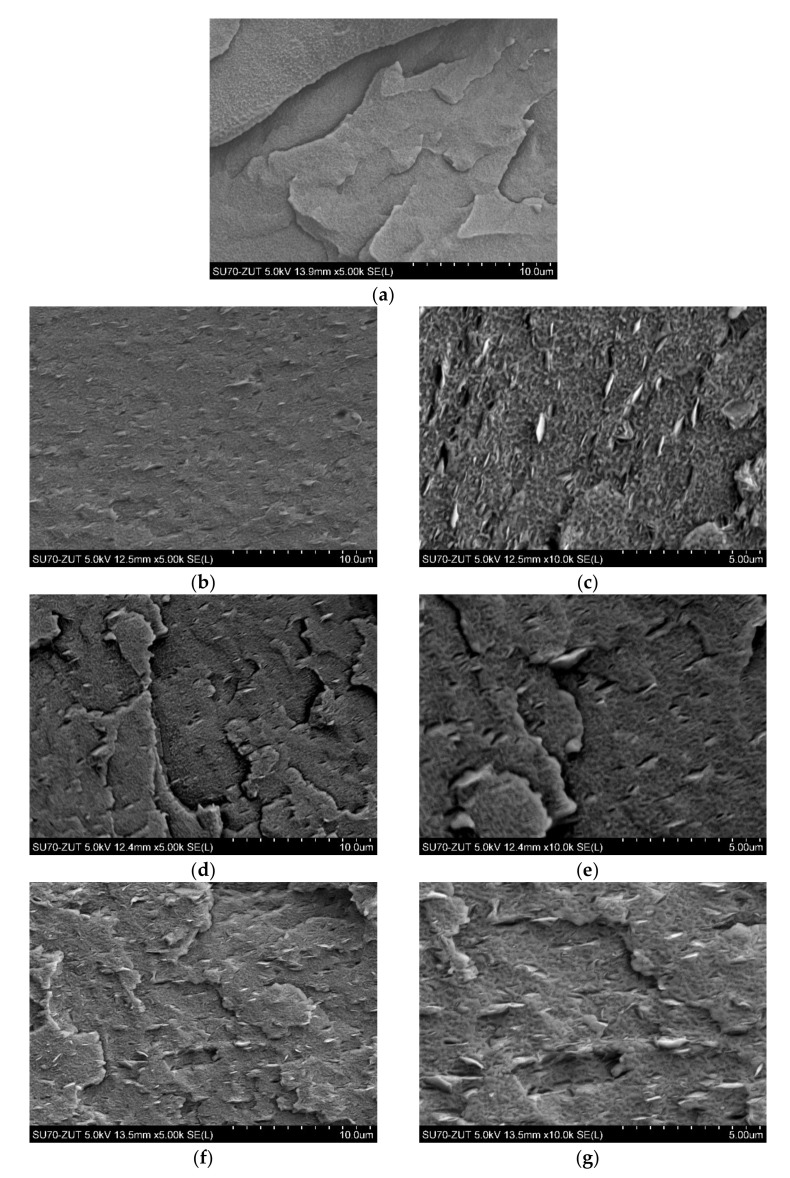

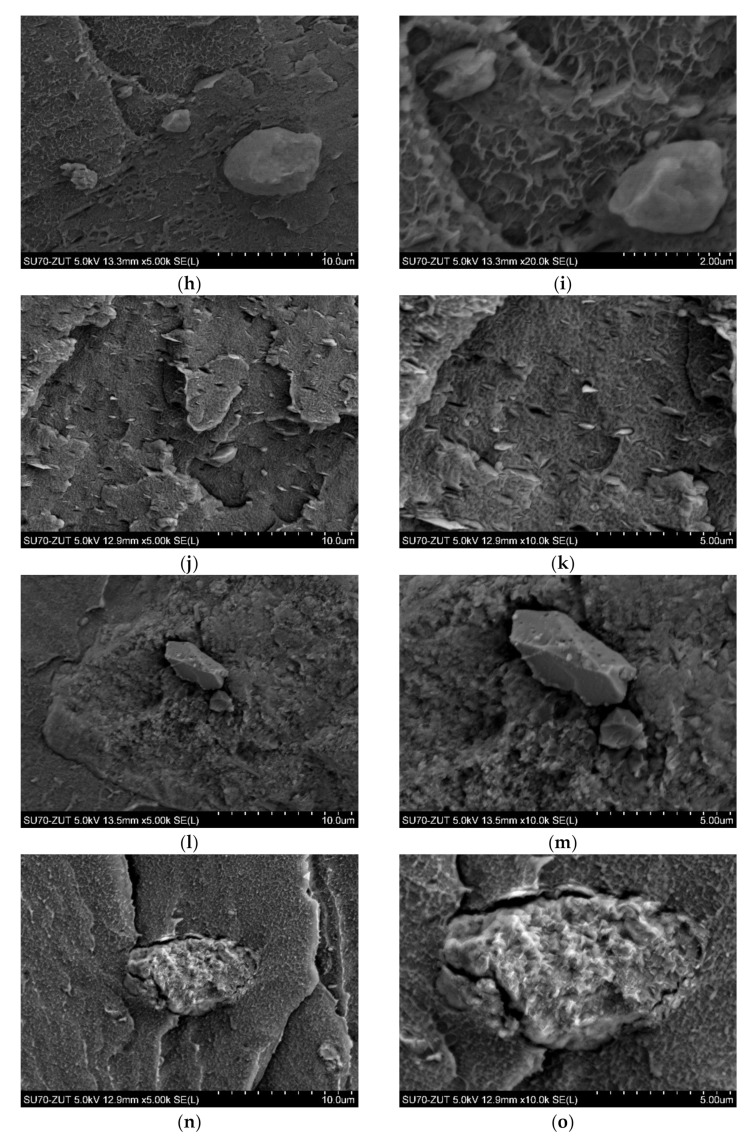

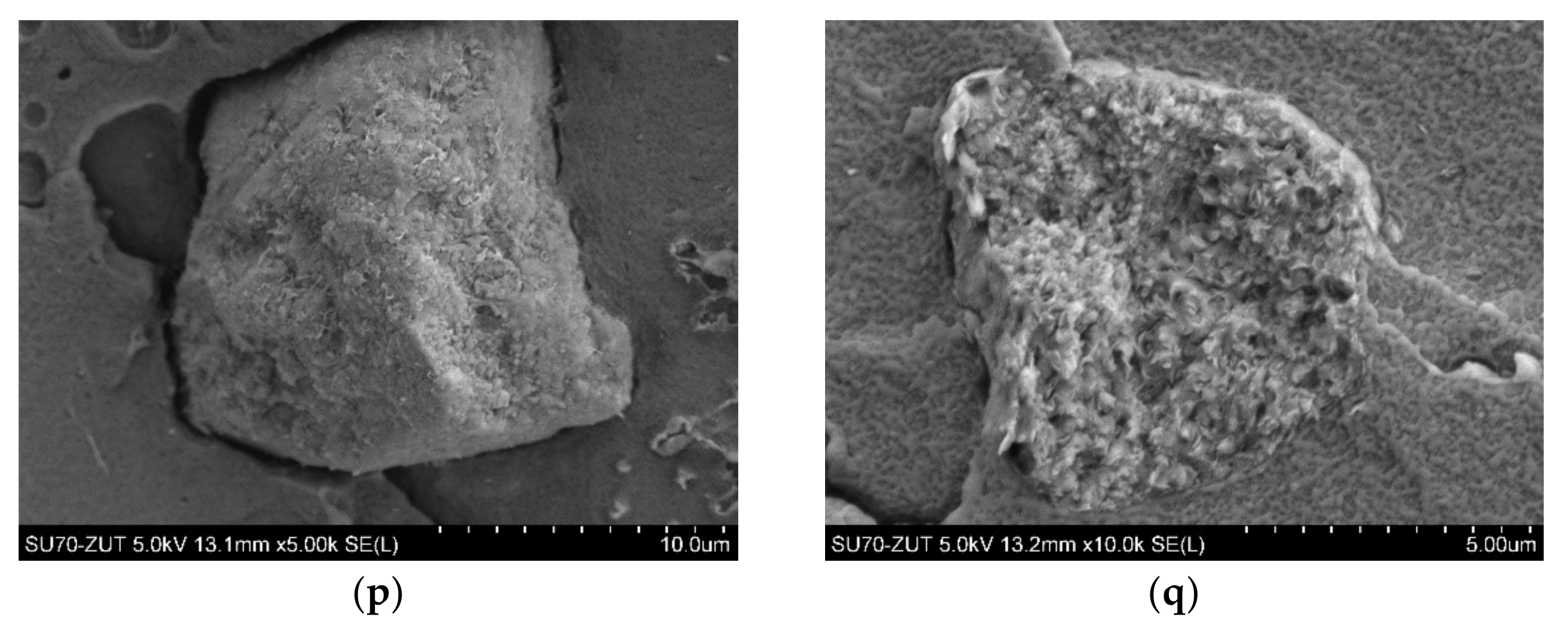
SEM micrographs: (**a**) PELS; (**b**,**c**) PELS 2.5% C20A; (**d**,**e**) PELS 5% C20A; (**f**,**g**) PELS 7% C20A; (**h**,**i**) PELS 5% C20A + HNT; (**j**,**k**) PELS 7% C20A + HNT; (**l**,**m**) PELS 2.5% HNT; (**n**,**o**) PELS 5% HNT; (**p**,**q**) PELS 7% HNT.

**Figure 4:**
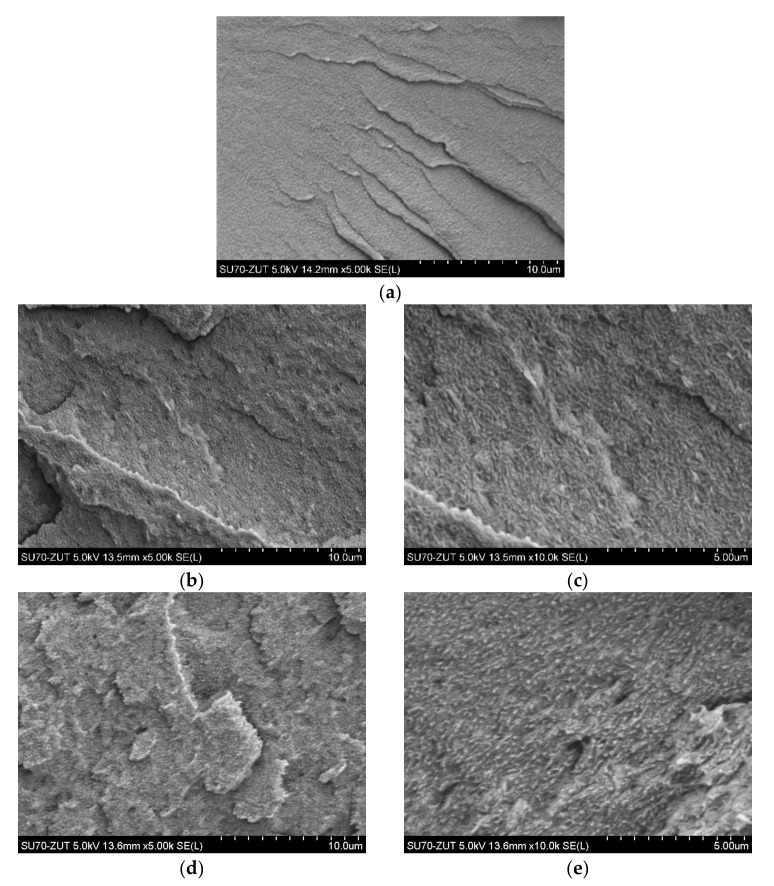

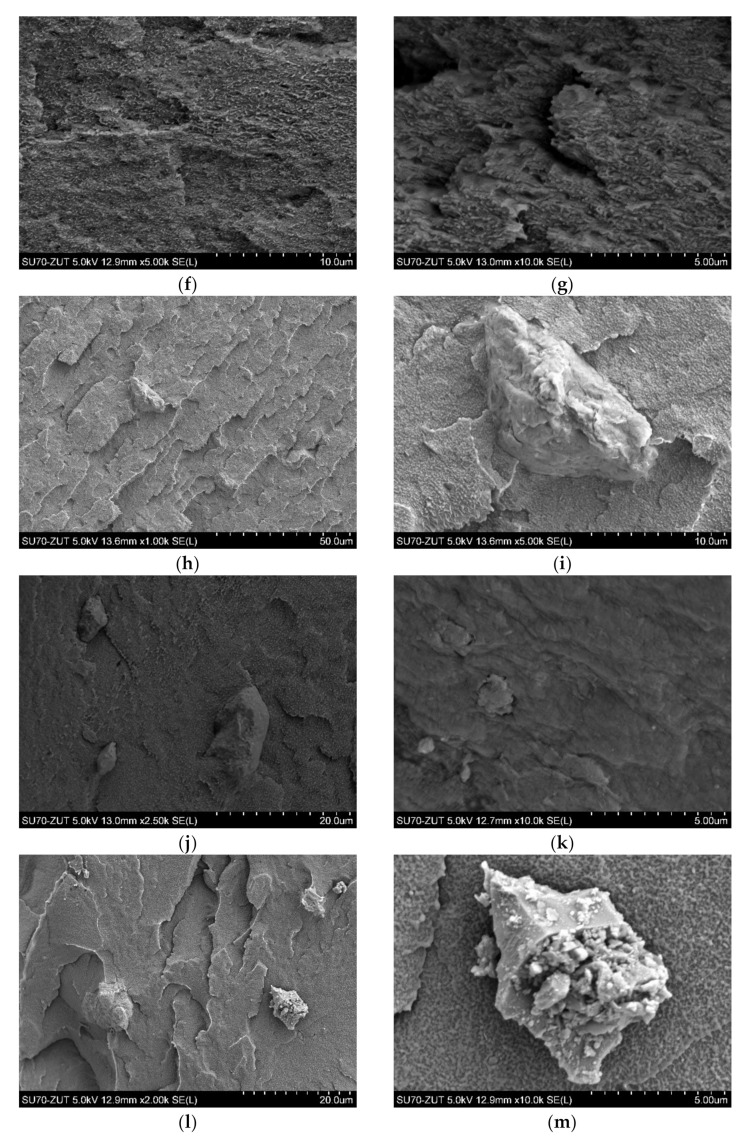

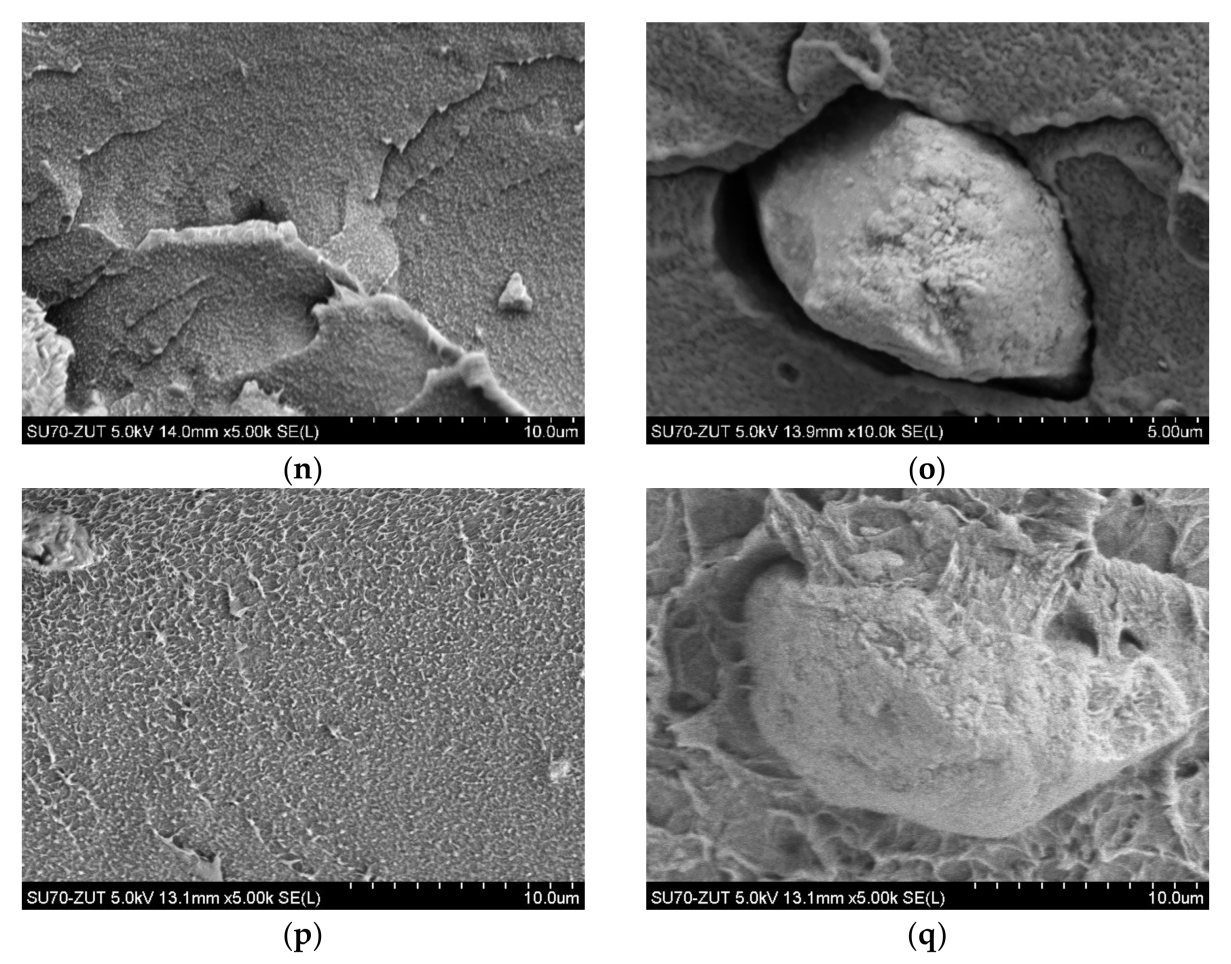
SEM micrographs: (**a**) PELC; (**b**,**c**) PELC 2.5% C20A; (**d**,**e**) PELC 5% C20A; (**f**,**g**) PELC 7% C20A; (**h**,**i**) PELC 5% C20A + HNT; (**j**,**k**) PELC 7% C20A + HNT; (**l**,**m**) PELC 2.5% HNT; (**n**,**o**) PELC 5% HNT; (**p**,**q**) PELC 7% HNT.

The morphology of different weight percentages (ranging from 0–7.0 wt.%) of C20A and HNTs nanoparticles within the PELS and PELC matrixes, as well as the hybrid system of nanofillers, were depicted in Figure 3 and Figure 4. Figure 3a and Figure 4a show the morphology of neat PELS and PELC, respectively, which is obvious that in the absence of nanofillers, the surfaces of both polymers are uniform, continuous, and smooth. Moreover, Figure 3b–g depict the morphology of PELS/C20A nanocomposites. From the Figures, it can be seen that at 2.5 wt.% to 7 wt.% of C20A, the nanoparticles were well dispersed in the matrix; however, the nanoplatelets were pulled out from the PELS polymer, which resulted from the preparation procedure, i.e., cryofracturing. In fact, there are weak interactions between polymer and nanoparticles. Figure 3h–k show the morphology of PELS/C20A/HNTs hybrid nanocomposites. In these hybrid nanocomposites, a good dispersion of C20A platelets with locally observed agglomerations of HNTs was observed. Furthermore, the morphology of PELS/HNTs nanocomposites combined with 2.5 wt.%, 5.0 wt.%, and 7.0 wt.%. were demonstrated in Figure 3l–q. In these specimens, only locally visible agglomerations were observed, in which the size of agglomerated particles was larger along with increasing the amounts of HNTs from 2.5 wt.% to 7 wt.%. Figure 4b–g show the morphology of PELC/C20A nanocomposites. In these samples, the C20A nanoparticles were well distributed within the PELC matrix, and there is no visible agglomeration of nanoplatelets, even at a higher amount of C20A. Figure 4h–k depict the morphology of PELC/C20A/HNT hybrid nanocomposites. In these nanocomposites, HNTs’ agglomeration was observed in the matrix. In fact, the presence of these agglomerations constitutes declining in the mechanical properties (discussed in Point 3.3). Figure 4l–q show the morphology of PELC/HNTs nanocomposites. In all of the PELC/HNTs nanocomposites, HNTs were not well dispersed in the PELC. The observations made for HNTs-containing nanocomposites in both PELS and PELC are in the agreement with the paper of Ravichandran G. et al. [26] who suggested ascertaining the necessity for limiting the nanofiller HNTs to 3 wt.%, beyond which critical evidence of poor interfacial interaction with the host epoxy matrix was recorded and more initial brittle cracking which constitutes declining of the mechanical properties was reported. In turn, when the concentration of HNTs was lower (1–2 wt.%) they observed uniform dispersion and distribution of HNTs within the epoxy matrix.

### 3.2. Structural Characterization of Polymer Nanocomposites

**Figure 5:**
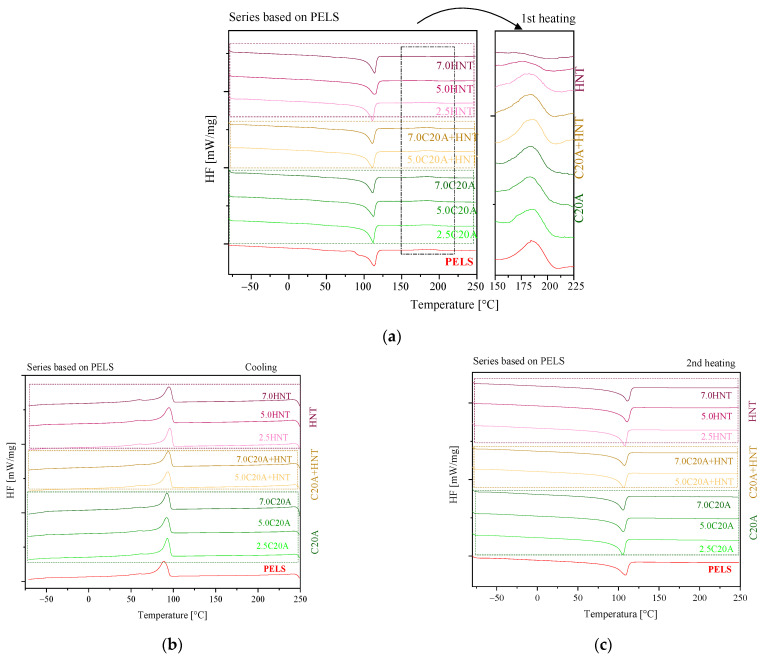
DSC thermograms recorded during first heating: (**a**) first heating; (**b**) cooling; (**c**) second heating for the series of nanocomposites based on PELS.

**Figure 6:**
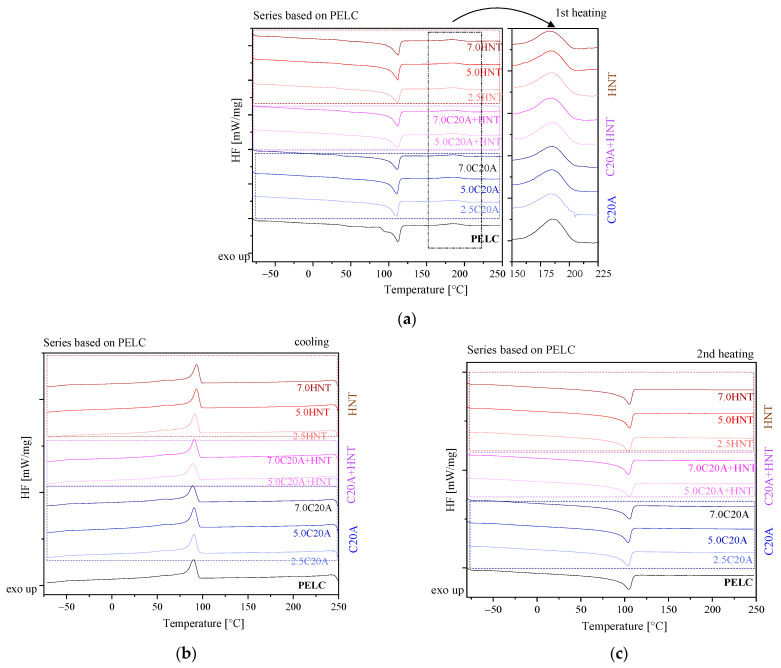
DSC thermograms recorded during first heating: (**a**) first heating; (**b**) cooling; (**c**) second heating for the series of nanocomposites based on PELC.

To compare the influence of mineral nanofillers on the phase transition temperatures of two types of insulation materials, one used DSC analysis. The DSC thermograms of the PELS-based nanocomposites are shown in Figure 5a–c, wherein Figure 6a–c represent the thermograms for PELC. Additionally, Table 1 and Table 2 summarized the values of the phase transition temperatures and the estimated values of degrees of crystallinity for individual composites. When analyzing DSC thermograms during the first heating for a neat polymer (PELS) (Figure 5a), one can observe a melting peak at 114 °C and the peak corresponding to cross-linking at 184.4 °C. In order to better present the cross-linking process, the scale in the temperature range of 150–225 °C has been enlarged. One can observe the crystallization temperature (T_c_) on the exotherm recorded during cooling from the melt at 88.7 °C. In turn, on the endotherm (from the second heating cycle), the melting temperature (Tm) is observed at 108 °C. The addition of the HNTs makes the cross-linking peak shift toward lower temperatures. For the nanocomposite with the highest content of HNTs, this peak is practically unseen. The addition of C20A (at all wt.% concentrations) to the matrix caused the decrease of the value of the melting temperature by ca 3 °C. In turn, for nanocomposites containing HNTs and hybrid nanocomposites, the influence of nanofillers was not observed. The changes in the melting temperature differ from one another by about 1 °C. The addition of nanofillers increases the value of crystallization temperature for all PELS-based nanocomposites by ca. 2–6 °C. For PELS-based nanocomposites, the addition of C20A causes a decrease in the degree of crystallinity, while the addition of HNTs increases slightly this value.

When analyzing DSC thermograms during the first heating cycle for a neat polymer (PELC) (Figure 6a), a melting peak at 112 °C and the peak corresponding to cross-linking at 185.2 °C are visible. On the exotherm recorded during cooling from the melt, one can observe Tc at 89.8 °C. In turn, on the endotherm recorded during the second heating cycle, the melting temperature (Tm) is observed at 104.6 °C. In the case of the cross-linking temperature, the addition of nanoparticles caused a decrease in the cross-linking temperature if compared to the neat polymer. As for the influence of nanofillers on the melting temperature, it is negligible. The addition of nanofillers increases the crystallization temperature for all PELC-based nanocomposites. When analyzing the value of the degree of crystallinity, it can be seen that the addition of HNTs and the hybrid system of HNTs/C20A into PELC, caused the degree of crystallinity to increase. However, along with the increase in the HNTs’ content, an explicit decrease in the degree of crystallinity was observed. In turn, the incorporation of C20A caused no significant changes in the values of the degree of crystallinity. Thomas et al. [27] discussed DSC results of XLPE and compared virgin compound with nano clay fillers with similar results indicating a comparably low effect of fillers on XLPE phase transformations. Liu [28] has compared LDPE and XLPE nanocomposites with MA, MH, MMT, and OMT, showing that DSC peaks do not change significantly with the addition of such additives, e.g., nanoclay montmorillonites.

**Figure 7:**
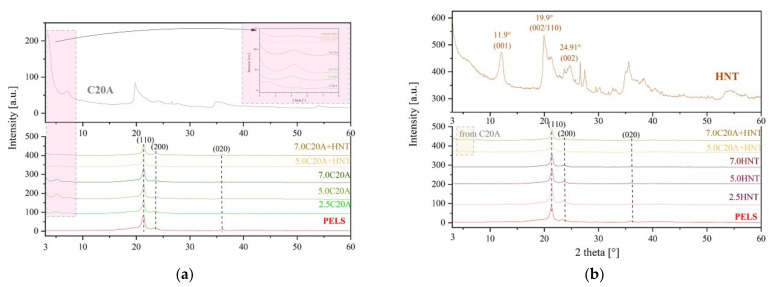
XRD patterns for the series of nanocomposites based on PELS: (**a**) in reference to C20A; (**b**) in reference to HNT.

**Figure 8:**
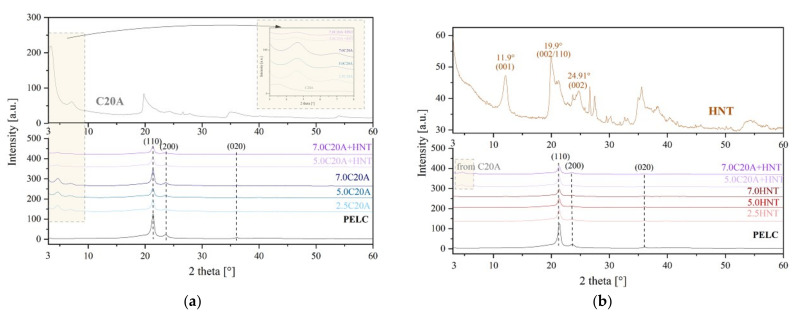
XRD patterns for the series of nanocomposites based on PELC: (**a**) in reference to C20A; (**b**) in reference to HNT.

X-ray diffraction (XRD) was used to characterize the layered structure of the PELS-based and PELC-based nanocomposites. Figure 7 shows the XRD patterns of the organoclay and PELS-based nanocomposites in reference to C20A (a) and HNT (b), while Figure 8 shows the XRD patterns of the organoclay and PELC-based nanocomposites in reference to C20A (a) and HNT (b). The PELS-based and PELC-based nanocomposites display a sharp peak located at 2θ of 21° (0.43 nm) and a smaller peak located at 2θ of 23° (0.39 nm). With increasing nanofillers’ content in composite, both peaks become smaller. The additional graph has been inserted to better observe the changes in the structure of clay-containing nanocomposites. It may be observed that the C20A organoclay shows two characteristic diffraction peaks: one at 2θ at ca. 7.0°, corresponding to a d_(002)_ basal spacing of 1.24 nm, and the other at 2θ at ca. 3.6°, corresponding to a d_(001)_ basal spacing of 2.45 mm. The distance, corresponding to the d_001_ plane, is termed the gallery spacing of the clay and is dependent on the modification. The (001) basal-plane spacing (d_001_) of the C20A (as received) is found to be 2.45 nm (peak at ca. 3.6°). For PELS-based nanocomposites, the (001) interlayer distance calculated from the XRD reflections decreased to ~1.69 nm, but for PELC-based distance calculated from the XRD, reflections decreased to ~1.87 nm. The decrease in the gallery spacing of the C20A in composites indicates that the clay platelets have not been intercalated by the polymer. Kavitha et al. [24] studied the dispersion of the organo-modified nanoclay in the XLPE nanocomposites matrix using X-ray diffraction, receiving good exfoliation for 2.5% and 5% additives and slight agglomeration confirmed by a small peak at 2θ of 6.7 for 7.5% and 10% of nanoclay composites. This may indicate that we have correctly selected the concentration of additives not exceeding 7% of the maximum share in XLPE compound. 

### 3.3. Mechanical Properties

**Figure 9:**
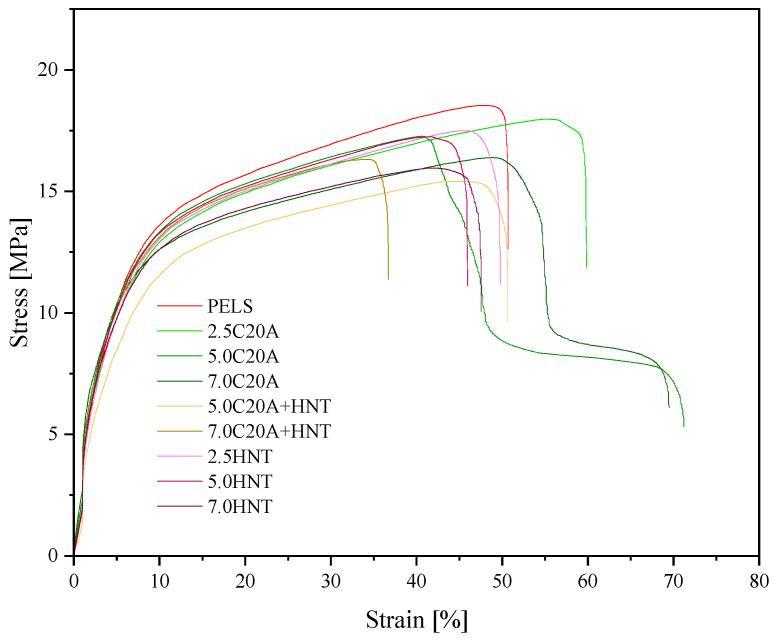
Representative stress–strain curves for the series of nanocomposites based on PELS.

**Figure 10:**
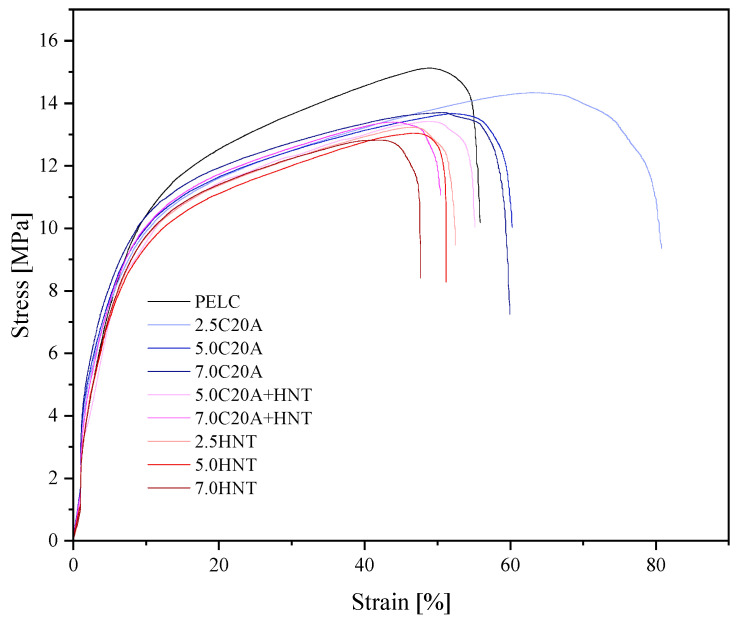
Representative stress–strain curves for the series of nanocomposites based on PELC.

Figure 9 shows representative stress–strain curves for PELS and PELC-based (hybrid) nanocomposites containing C20A and HNT, whereas Figure 10 shows representative stress-strain curves for PELS and PELC-based (hybrid) nanocomposites containing C20A and HNT. Table 3 summarizes the tensile parameters determined during the measurement, i.e., Young′s Modulus (calculated from strain 0.05% to 0.25%), tensile strength and elongation at yield, strength and elongation at break, as well as hardness and density for PELS, whereas Table 4 presents the same parameters for PELC. Comparing both neat polymers with each other, it can be seen that PELS has better mechanical properties than PELC, for instance, Young’s modulus value for PELS is greater by almost 100% in comparison to PELC, which makes it more promising for the future applications. By analyzing the obtained data for PELS, one can conclude that the addition of 7.0 wt.% of C20A improved the mechanical properties of the polymer matrix, increasing Young′s modulus value by 1.5 times. In turn, the value of elongation at yield was first improved at the addition of 2.5% C20A, while the higher content did not make any difference in the values of εy. Moreover, the addition of C20A causes an improvement in both, strength and elongation at break of PELS, and causes no change in the value of hardness. As for the density, as expected, the higher the filler content, the higher the density, resulting from the rule of a mixture of two materials with different densities. In turn, the addition of HNTs caused the deterioration of tensile properties, when comparing nanocomposites to the neat polymer. Only at the concentration of 7.0 wt.% of HNTs, one could observe an improvement in the value of Young′s modulus. Moreover, the addition of HNTs caused only a small effect on the changes in hardness and density. Moreover, in the case of hybrid nanocomposites based on PELS, the mechanical properties were deteriorated in comparison to the neat polymer matrix. Such a decrease in mechanical properties in the case of PELS/HNTs nanocomposites may be due to the agglomeration of particles, observed on SEM micrographs. In the case of PELC-based nanocomposites, one could have drawn similar observations regarding mechanical properties, i.e., the addition of C20A had a positive effect on the improvement of mechanical properties, while the addition of HNTs worsened these properties in relation to neat polymer due to poor dispersion in the whole volume of the polymer matrix. However, for PELC-based nanocomposites, the addition of nanoparticles caused an increase in the values of hardness and density. Pleşa et al. [29] studied the mechanical properties of epoxy resin-based micro nanocomposites, showing that the tensile strength of micro-filled composites has decreased by increasing filler content, which is related to debility of polymer base matrix, like it has a place in terms of XLPE composites presented in this paper. Lim et al. [30] showed a variation of Young’s modulus versus ZnO, Al_2_O_3_, and OMMT nanofillers loading in the XLPE nanocomposites, where it has increased with the filler concentration. In our study, we have observed the same tendency with C20A, whilst the HNT additive has reduced elastic modulus, which is related to its nanostructure and the morphology. Polanský et al. [31] described the toughening of LDPE by HNTs by the formation of an interphase region between the matrix and the nanoparticles, limiting the orientation and alignment of polymer chains that determine the reduction in tensile strength and elongation on break, observing a similar effect.

### 3.4. Utilitarian Properties

#### 3.4.1. Water Absorption

**Figure 11:**
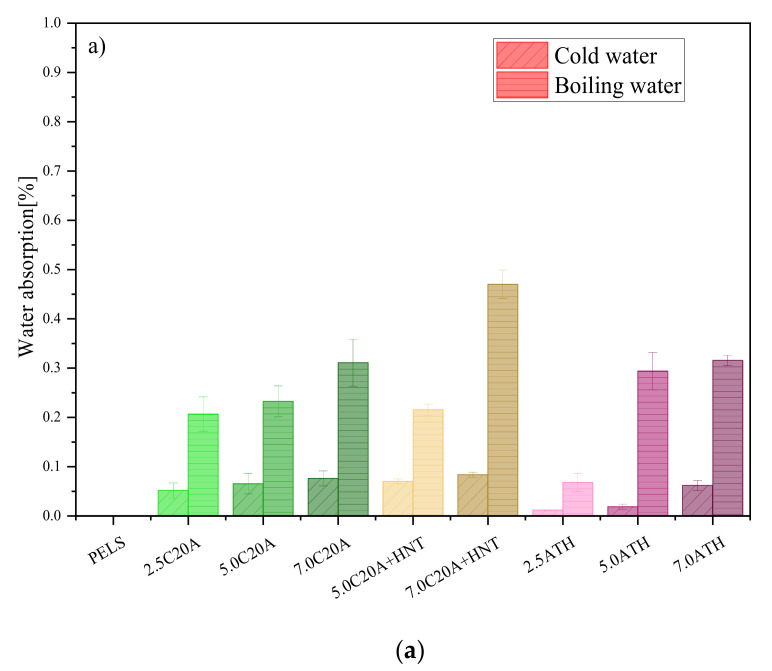

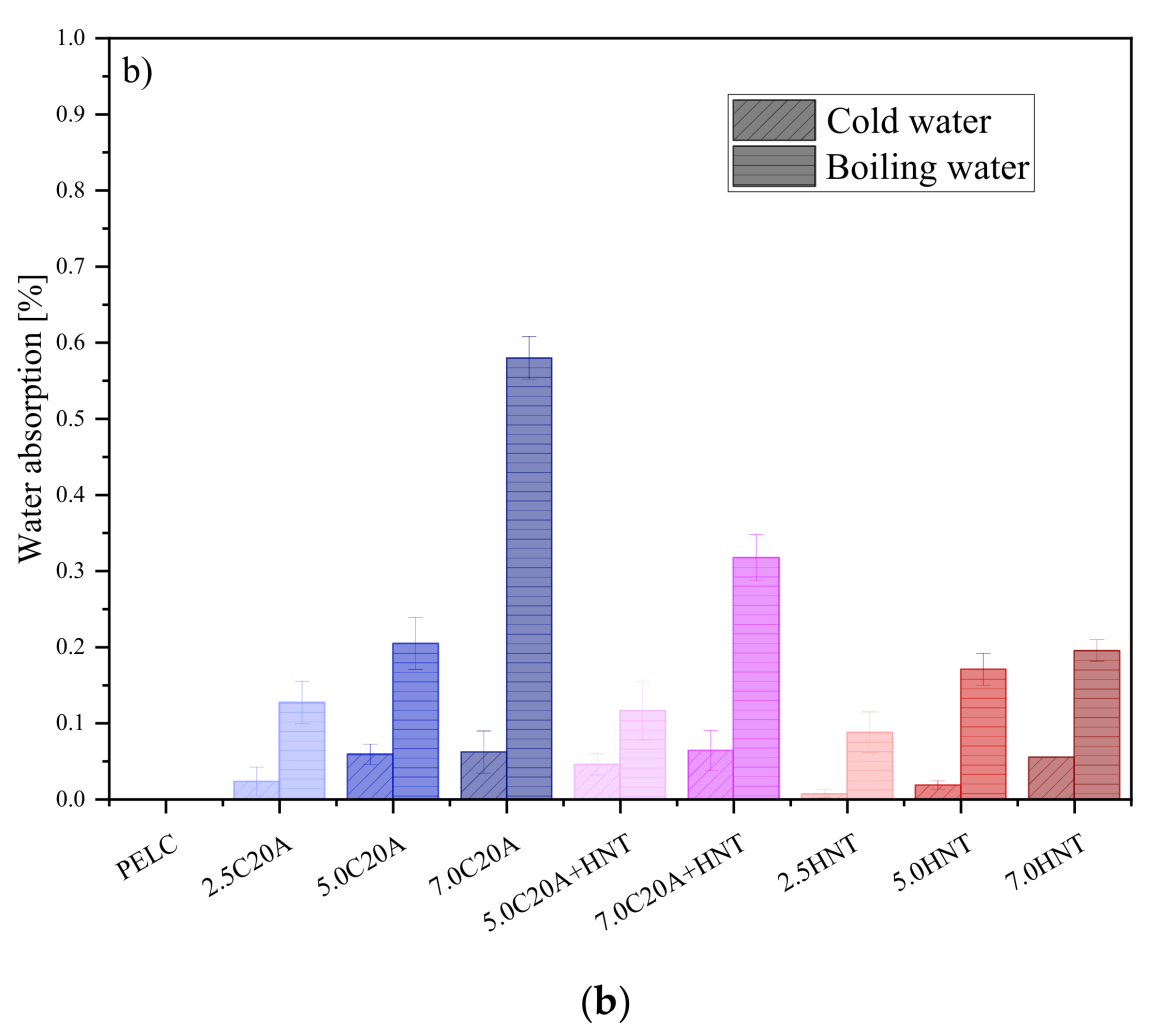
Cold and hot water absorption: (**a**) PELS-based nanocomposites; (**b**) PELC-based nanocomposites.

Figure 11 shows the water absorption of the tested materials. The measurements were carried out in both cold and boiling water. As for the neat materials, one can see that both PELS and PELC do not absorb water (WA = 0%). The addition of nanofillers caused an increase in the value of WA for all nanocomposites. The nanocomposites with the lowest concentrations of nanofillers have the lowest values of WA, while an increasing nanoparticles’ concentration results in higher WA values. In the case of the boiling water test for PELS-based nanocomposites, it can be seen that both nanofillers i.e., C20A and HNTs affect the water absorption to the same extent. However, in the cold-water test, the HNTs absorbed significantly less water than the C20A. The hybrid with the highest concentration of nanofillers achieved the highest WA value of all PELC-based nanocomposites. For PELC-based nanocomposites, nanocomposites with a concentration of 2 and 5 wt.%, have similar values to nanocomposites with HNTs. On the other hand, the nanocomposite with the highest concentration of C20A achieved the WA value, almost three times higher than the other nanocomposites. Perthué et al. [32] studied PE/ATH composites used in cable applications in terms of effects of the thermo-oxidation on water absorption. One of the conclusions is that the presence of hydrophilic fillers in the polymer matrix prevents the diffusion of water into the polymer matrix. Qingyue et al. [33] mentioned typical layered silicate such as OMMT with a large specific surface area in terms of forming ionic bond with the matrix resin such as XLPE. The intercalated OMMT restricted the diffusion of water molecules, increasing the water tree resistance of the nano-composite. Studies have shown that such an additive like MMT has a positive influence on reducing water trees′ length and shape.

#### 3.4.2. TGA

**Figure 12:**
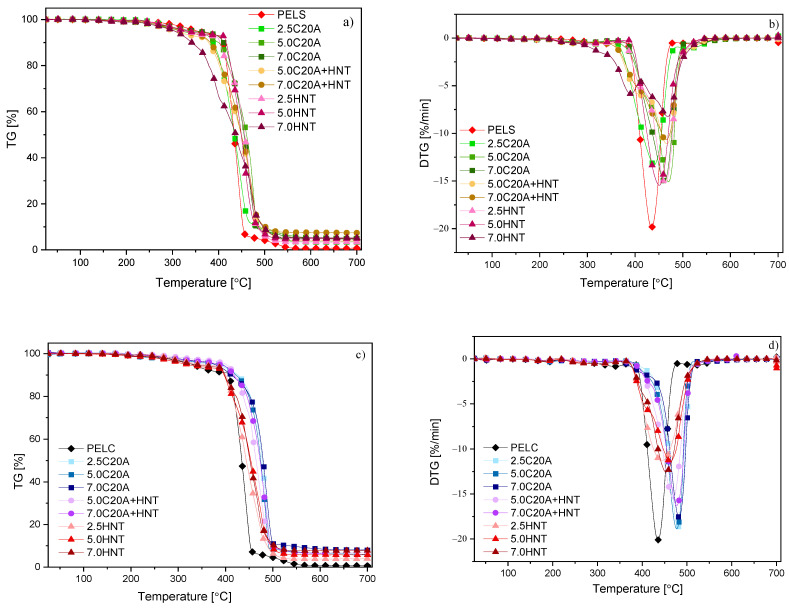
Thermo-oxidative stability: (**a**,**b**) PELS-based nanocomposites; (**c**,**d**) PELC-based nanocomposites.

One of the parameters, that can limit the applications of the insulation materials, is their thermal stability. The thermo-oxidative stability of the XLPE-based nanocomposites with the addition of clay was studied, and the TG and DTG curves with different contents of PELS-based nanocomposites are presented in Figure 12a,b and PELC-based nanocomposites (c,d). Additionally, the characteristic temperatures of 5, 10, 50, and 90% of weight loss (T_5%_,T_10%_,T_50%_,T_100%_), in an oxidizing atmospheres are summarized in Table 5. The addition of nanoparticles decreases the value of T_5%_ for all PELS-based nanocomposites compared to neat polymer. The addition of nanofillers caused an increase in the values of T_50%_ and T_90%_ by 20–40 °C compared to a neat polymer, except for the highest concentration of HNTs, where the value is the same as for the neat polymer. When analyzing mass residue at 700 °C, one can see that the values are the sum of the residual carbon residues and the filler concentration in individual nanocomposites. The value of T_5%_ increased by as much as 50 °C for the PELC-based nanocomposite with the addition of C20A compared to the neat polymer. In turn, with an increase in the concentration of HNTs, the value of\T_5%_ decreased. One can notice the same dependencies in the case of the T_10%_ value. For T_50%_ and T_90%_, the addition of nanofillers caused an increase in their values by 30–50 °C compared to the neat polymer. A similar observation on the improvement in thermo-oxidative stability was made by Peila [34] who showed that the presence of nanoclays such as commercially available Cloisite had a clear effect on the thermal stability and thermo-oxidative properties of the LDPE-based composites. Moreover, Polanský [31] indicates the phenomenon of improving heat dissipation from the polymer structure through the addition of nanofiller in the form of halloysite nanotubes, which results in improved thermal stability.

#### 3.4.3. Volume Resistivity

The volume resistivity measurement was performed to check if the incorporation of hydrophilic minerals does not deteriorate the properties of the polymer matrices (PELS and PELC) intended for isolation in submarine power cables. It can be seen that despite the incorporation of nanofillers, all nanocomposites were found to be electrically resistant (Table 6). The neat PELS has a higher volume resistivity than neat PELC. In the case of PELS-based nanocomposites, it is visible that the addition of nanofillers caused a slight decrease in the value of the volume resistivity in relation to the neat polymer (one order of magnitude). However, in the case of PELC-based nanocomposites, only the addition of C20A causes a slight decrease in the value of volume resistivity, while in the case of hybrids and HNTs-containing nanocomposites, one can notice an increase in the value of volume resistivity. The observed changes in the values of volume resistivity as a function of both, the type of polymer matrix and the incorporated nanofillers are generally within 2–3 orders of magnitude and confirm that all analyzed materials were non-conductive. These observations are promising from the point of view of future applications of the analyzed systems.

## 4. Conclusions

The incorporation of C20A and HNT into XLPE caused an improvement in their mechanical properties. Comparing both series of nanocomposites, the one based on homopolymer (PELS) with addition of C20A indicated the greatest potential in terms of mechanical properties improvement. HNTs show a greater tendency to agglomerate and thus show worse dispersion in XLPE matrixes, especially at higher concentrations (5 wt.% 7.0 wt.%). In turn, C20A has a greater ability to be dispersed evenly in the whole volume of polymer regardless of the concentration. In the case of both series of nanocomposites, the influence of nanofillers on the changes in thermal properties (based on DSC analysis) is negligible. Due to the hydrophilic nature of nanofillers, the composites show an increase in values of water absorption which causes the phenomenon of moisture trapping, which could potentially reduce the initiation of undesirable water treeing effect. It may be assumed that since C20A increases the moisture barrier effect along with mechanical properties of XLPE in a stronger manner, one can find nanocomposites with its content as the most promising among tested composites in terms of cable insulation resistance in water treeing phenomenon.

## Figures and Tables

**Table 1:** Thermal properties of PELS-based nanocomposites.

Material	Tc [°C]	ΔHc [J/g]	Tm [°C]	ΔHm [J/g]	Tcl [°C]	ΔHcl [J/g]
PELS	88.7	116.8	108.4	118.3	184.4	9.44
PELS/2.5 C20A	92.9	119.4	105.4	112.2	185.0	8.62
PELS/5.0 C20A	92.0	114	105.8	110.8	184.9	8.06
PELS/7.0 C20A	92.6	112.1	105.8	108.3	184.9	9.01
PELS/5.0 C20A + HNT	93.7	117.6	106.5	116.3	186.1	9.79
PELS/7.0 C20A + HNT	94.0	110.9	107.1	105.2	184.6	8.34
PELS/2.5HNT	95.8	123.0	107.9	119.7	184.4	6.38
PELS/5.0HNT	96.5	120.9	109.0	121.7	176.7	2.47
PELS/7.0HNT	96.8	120.1	109.7	114.3	-	-

Tc, ΔHc, crystallization temperature and the corresponding enthalpy of crystallization; Tm, ΔHm, melting temperature and the corresponding enthalpy of melting Tcl, ΔHcl–cross-linking temperature and the corresponding enthalpy of cross-linking (determined from the first heating scan).

**Table 2:** Thermal properties of PELC based nanocomposites.

Material	Tc [°C]	ΔHc [J/g]	Tm [°C]	ΔHm [J/g]	Tcl [°C]	ΔHcl [J/g]
PELC	89.8	111.0	104.6	101.5	185.2	18.07
PELC/2.5C20A	90.3	106.0	103.8	103.8	184.2	13.55
PELC/5.0 C20A	90.6	114.3	104.0	99.57	184.2	14.01
PELC/7.0 C20A	89.1	101.9	105.4	101.3	184.1	12.87
PELC/5.0 C20A + HNT	89.6	105.9	105.3	122.5	185.2	14.59
PELC/7.0 C20A + HNT	91.0	104.7	104.3	99.86	184.5	14.47
PELC/2.5HNT	91.3	108.5	104.3	103.2	184.7	14.90
PELC/5.0HNT	93.2	107.0	105.6	122.9	183.9	12.41
PELC/7.0HNT	93.2	104.0	105.5	96.46	182.3	10.42

Tc, ΔHc, crystallization temperature and the corresponding enthalpy of crystallization; Tm, ΔHm, melting temperature and the corresponding enthalpy of melting Tcl, ΔHcl–cross-linking temperature and the corresponding enthalpy of cross-linking (determined from the first heating scan).

**Table 3:** Mechanical properties for PELS-based nanocomposites.

Material	E [MPa]	$\sigma_{y}$ [Mpa]	$\varepsilon_{y}$ [%]	$\sigma_{b}$ [Mpa]	$\varepsilon_{b}$ [%]	Hardness [ShD]	Density $\left(g/cm^{3}\right)$
PELS	199 ± 15.8	18.5 ± 0.2	48.3 ± 3.7	1.2 ± 0.1	50.6 ± 4.3	45.0 ± 0.3	0.929 ± 0.001
PELS/2.5C20A	223.3 ± 17.3	17.9 ± 1.4	55.0 ± 4.5	1.8 ± 0.1	59.8 ± 4.6	45.0 ± 0.2	0.943 ± 0.003
PELS/5.0 C20A	206.9 ± 16.9	17.2 ± 0.4	40.6 ± 2.8	2.7 ± 0.1	71.3 ± 6.5	45.0 ± 0.3	0.959 ± 0.002
PELS/7.0 C20A	335.6 ± 22.5	16.3 ± 0.5	49.0 ± 1.5	2.7 ± 0.2	69.6 ± 3.3	45.0 ± 0.3	0.960 ± 0.001
PELS/5.0 C20A + HNT	177.7 ±11.2	15.4 ± 0.6	44.0 ± 3.6	2.8 ± 0.1	50.7 ± 4.6	45.0 ± 0.5	0.956 ± 0.002
PELS/7.0 C20A + HNT	181.3 ± 10.4	16.2 ± 0.4	32.8 ± 1.9	1.9 ± 0.1	36.7 ± 2.8	45.0 ± 0.2	0.992 ± 0.006
PELS/2.5HNT	160.6 ± 12.6	17.5 ± 0.1	45.9 ± 2.5	3.3 ± 0.2	49.8 ± 2.2	46.0 ± 0.5	0.945 ± 0.002
PELS/5.0HNT	181.6 ± 13.3	17.2 ± 0.2	41.6 ± 2.8	2.8 ± 0.1	46.0 ± 3.8	47.0 + 0.5	0.963 ± 0.002
PELS/7.0HNT	213.9 ± 15.2	16.0 ± 0.6	42.5 ± 3.3	2.8 ± 0.1	47.5 ± 4.3	47.0 + 0.3	0.986 ± 0.002

E—Young’s Modulus (calculated from strain 0.05% to 0.25%); *σ_y_*—tensile strength at yield, *σ_b_*, *ε_b_*—strength and elongation at break respectively.

**Table 4:** Mechanical properties for PELC-based nanocomposites.

Material	E [Mpa]	$\sigma_{y}$ [Mpa]	$\varepsilon_{y}$ [%]	$\sigma_{b}$ [Mpa]	$\varepsilon_{b}$ [%]	Hardness [ShD]	Density $\left(g/cm^{3}\right)$
PELC	102.1 ± 9.8	15.1 ± 0.7	49.2 ± 3.9	2.3 ± 0.1	56.1 ± 4.4	39.0 ± 0.5	0.934 ± 0.004
PELC/2.5C20A	155.0 ± 11.2	14.3 ± 0.1	63.4 ± 3.0	2.4 ± 0.2	81.4 ± 7.0	40.0 ± 0.5	0.951 ± 0.026
PELC/5.0 C20A	153.0 ± 12.4	13.6 ± 0.3	52.3 ± 4.4	2.7 ± 0.1	60.5 ± 3.7	43.0 ± 0.5	0.958 ± 0.011
PELC/7.0 C20A	152.1 ± 14.7	13.6 ± 0.2	48.5 ± 1.8	2.5 ± 0.2	60.2 ± 2.1	43.0 ± 0.5	0.963 ± 0.003
PELC/5.0 C20A + HNT	127.8 ± 8.7	13.1 ± 0.3	49.2 ± 3.6	2.3 ± 0.1	55.2 ± 4.8	43.0 ± 0.3	0.953 ± 0.004
PELC/7.0 C20A + HNT	149.7 ± 10.3	13.4 ± 0.2	44.2 ± 2.1	1.9 ± 0.1	50.5 ± 3.6	43.0 ± 0.3	0.968 ± 0.005
PELC/2.5HNT	79.5 ± 6.5	13.2 ± 0.4	47.3 ± 4.2	1.3 ± 0.1	52.5 ± 4.1	43.0 ± 0.4	0.948 ± 0.002
PELC/5.0HNT	116 ± 10.7	13.0 ± 0.4	49.1 ± 3.5	2.3 ± 0.2	56.7 ± 5.1	43.0 + 0.5	0.955 ± 0.026
PELC/7.0HNT	99.4 ± 7.2	12.8 ± 0.4	42.1 ± 2.7	2.8 ± 0.2	62.8 ± 5.3	44.0 + 0.3	0.961 ± 0.008

E—Young’s Modulus (calculated from strain 0.05% to 0.25%); *σ_y_*—tensile strength at yield, *σ_b_*, *ε_b_*—strength and elongation at break respectively.

**Table 5:** Thermo-oxidative stability parameters for PELS-based and PELC-based nanocomposites.

Material	T_5%_ [°C]	T_10%_ [°C]	T_50%_ [°C]	T_90%_ [°C]	R (700 °C) [%]	TDTG2 * [°C]	TDTG1 [°C]	TDTG2 [°C]
PELS	336	408	434	453	0.69	-	434	533
PELS/2.5 C20A	311	385	434	477	3.48	308	441	525
PELS/5.0 C20A	298	392	460	482	4.97	302	472	-
PELS/7.0 C20A	315	408	455	481	4.68	306	462	-
PELS/5.0 C20A + HNT	289	375	448	486	3.89	292	410/469	-
PELS/7.0 C20A + HNT	305	377	448	495	7.38	-	398/468	-
PELS/2.5HNT	311	401	456	482	3.37	295	412/466	-
PELS/5.0HNT	312	415	449	482	4.89	304	448	-
PELS/7.0HNT	336	408	434	453	6.09	-	434	533
PELC	315	403	434	452	0.65	-	435	530
PELC/2.5 C20A	333	420	477	495	5.98	-	479	-
PELC/5.0 C20A	348	418	474	491	6.01	-	479	-
PELC/7.0 C20A	365	407	479	501	8.02	-	484	-
PELC/5.0 C20A + HNT	388	416	464	491	6.79	309	471	-
PELC/7.0 C20A + HNT	364	414	472	484	7.85	-	478	-
PELC/2.5HNT	322	397	443	486	3.95	305	445	-
PELC/5.0HNT	315	397	453	494	5.77	303	460	-
PELC/7.0HNT	311	397	452	498	7.80	297	457	-

T_5%_, T_10%_, T_50%_, T_90%_—temperatures corresponding to 5, 10, 50 and 90% of mass loss, respectively; R (700 °C)% mass residue at 700 °C; TDTG1 and TDTG2 and TDTG2 * temperatures corresponding to the maximum of mass loss.

**Table 6:** Volume resistivity of PELS-based and PELC-based nanocomposites.

Material	Volume Resistivity ρ_s_ [Ω m]
PELS	336
PELS/2.5C20A	311
PELS/5.0C20A	298
PELS/7.0C20A	315
PELS/5.0C20A + HNT	289
PELS/7.0C20A + HNT	305
PELS/2.5HNT	311
PELS/5.0HNT	312
PELS/7.0HNT	336
PELC	315
PELC/2.5C20A	333
PELC/5.0C20A	348
PELC/7.0C20A	365
PELC/5.0C20A + HNT	388
PELC/7.0C20A + HNT	364
PELC/2.5HNT	322
PELC/5.0HNT	315
PELC/7.0HNT	311

Voltage U = 1000 V, T = 20 °C.

## Data Availability

The data presented in this study are available on request from the corresponding author. The data are not publicly available.
